# Auranofin Inhibits RANKL-Induced Osteoclastogenesis by Suppressing Inhibitors of *κ*B Kinase and Inflammasome-Mediated Interleukin-1*β* Secretion

**DOI:** 10.1155/2019/3503912

**Published:** 2019-04-22

**Authors:** Hyun Young Kim, Kyeong Seok Kim, Myung Ji Kim, Hyung-Shik Kim, Kwang-Youl Lee, Keon Wook Kang

**Affiliations:** ^1^College of Pharmacy and Research Institute of Pharmaceutical Sciences, Seoul National University, Seoul 08826, Republic of Korea; ^2^College of Pharmacy, Sungkyunkwan University, Suwon 16419, Republic of Korea; ^3^College of Pharmacy, Chonnam National University, Gwangju 61186, Republic of Korea

## Abstract

Osteoporosis is a degenerative metabolic disease caused by an imbalance between osteogenesis and osteoclastogenesis. Increased levels of proinflammatory cytokines combined with decreased estrogen levels, which are commonly seen in postmenopausal women, can lead to overactivation of osteoclasts. Therefore, targeting osteoclast maturation may represent a novel strategy for both treating and preventing osteoporosis. Auranofin is a gold-based compound first approved in 1985 for the treatment of rheumatic diseases. Here, we examined whether auranofin suppresses osteoclast differentiation *in vitro* and *in vivo*. Auranofin was shown to suppress receptor activator of NF-*κ*B ligand- (RANKL-) induced osteoclastogenesis in mouse bone marrow macrophages (BMMs) and Raw264.7 macrophages. Cotreatment of macrophages with auranofin blocked the RANKL-induced inhibitors of *κ*B kinase (IKK) phosphorylation, resulting in inhibition of nuclear translocation of p65. The pan-caspase inhibitor nivocasan potently reduced not only inflammasome-mediated interleukin-1*β* (IL-1*β*) secretion but also osteoclast differentiation in BMMs. Auranofin suppressed inflammasome activation, as evidenced by decreased production of cleaved IL-1*β* in both bone marrow-derived macrophages (BMDMs) and J774.A1 cells. Loss of both bone mass in ovariectomized mice was significantly recovered by oral administration of auranofin. Taken together, these data strongly support the use of auranofin for the prevention of osteoclast-related osteoporosis.

## 1. Introduction

Bone is a hard and rigid organ, though one that remains highly dynamic in nature, undergoing constant remodeling according to the balance between osteoblast and osteoclast activity. Osteoblasts make and fill the bone matrix, while osteoclasts absorb bone to aid new bone formation [1]. When the activity of osteoclasts exceeds that of osteoblasts, total bone mass in the body decreases, increasing the susceptibility to bone breaks in response to even small external impacts. Osteoporosis is a degenerative metabolic disease caused by an imbalance in skeletal remodeling [2]. Therapeutic agents capable of inhibiting osteoclast activity and differentiation have been proposed as a potential first-line treatment option for osteoporosis and may help increase existing bone mass.

In osteoclastogenesis, bone marrow cells migrate to the surface of the bone and differentiate into osteoclasts. Binding of receptor activator of nuclear factor-*κ*B (NF-*κ*B) ligand (RANKL) to receptor activator of NF-*κ*B (RANK) on the surface of osteoclasts plays a key role in this differentiation [3]. RANK signaling is triggered by cytoplasmic factors, leading to activation of at least five distinct signaling cascades, including inhibitor of NF-*κ*B kinase (IKK), c-Jun N-terminal kinase (JNK), p38 kinase, extracellular signal-regulated kinase (ERK), and Src, all of which are known to be activated in the course of osteoclast formation. Of those signaling cascades, only the role of NF-*κ*B is fully understood in osteoclast formation [3].

Denosumab is the first FDA-approved monoclonal antibody capable of directly targeting RANKL, resulting in decreased bone turnover and increased bone mineral density (BMD) [4]. Despite a higher annual treatment cost compared to other medications for osteoporosis, denosumab was found to be a cost-effective option in older osteoporotic male patients in the United States [5]. However, denosumab remains available only by injection, with no orally effective inhibitors of RANKL-dependent osteoclastogenesis yet available.

Auranofin is an orally available anti-rheumatoid arthritis (RA) agent composed of an elemental gold particle, Au(I), in complex with a sulfur-containing ligand. Administration of auranofin to RA patients has been shown to reduce levels of inflammatory cytokines, including interleukin-1 (IL-1) and tumor necrosis factor-*α* (TNF-*α*) [6]. Although the pharmacological mechanism underlying auranofin activity is not fully understood, the anti-inflammatory effects of auranofin are postulated to be related to inhibition of NF-*κ*B [7]. Because NF-*κ*B activity is a prerequisite not only for the transcription of proinflammatory genes but also differentiation of bone marrow cells to osteoclasts, we hypothesized that the anti-RA agent auranofin can prevent RANKL-induced osteoclast formation. Moreover, it has been reported that auranofin decreases osteoclastic bone resorption in bone slice assay [8]. In this study, we demonstrate that auranofin inhibits differentiation of macrophages to osteoclasts and prevents bone resorption both *in vivo* and *in vitro*. Our data further reveal that the antiosteoclastogenesis effect of auranofin is mediated through the inhibition of both IKK-mediated NF-*κ*B activation and inflammasome-dependent IL-1*β* secretion in bone marrow-derived macrophages (BMDMs).

## 2. Materials and Methods

### 2.1. Materials

Auranofin (BML-EI206-0100) was purchased from Enzo Life Sciences (Lausen, Switzerland). Primary antibodies against the following proteins were used in this study: cathepsin K (E-7), NFATc1 (7A6), and p65 were from Santa Cruz Biotechnology (Dallas, TX, USA). Specific antibodies against lamin A/C, IKK, I*κ*B*α*, and phospho-I*κ*B*α* (Ser32) were from Cell Signaling Technology (Danvers, MA, USA), and the antibody against IKK*β* was purchased from Abcam (Cambridge, MA, USA). Tartrate-resistant acid phosphatase (TRAP) staining kit (386A-1KT) and 3-(4,5-dimethylthiazole-2-yl)-2,5-dipenyltetrazolium bromide (MTT) were obtained from Sigma-Aldrich (St. Louis, MO, USA). Murine M-CSF was supplied from PeproTech (Rocky Hill, NJ, USA).

### 2.2. *In Vitro* Osteoclastogenesis Assay

Murine osteoclasts were prepared from bone marrow cells of C57BL/6 mice. Bone marrow cells were plated in petri dishes containing *α*-minimum essential medium, 10% fetal bovine serum with macrophage colony-stimulating factor (M-CSF; 30 ng/mL) for 3 days. Floating cells were removed, and those that became adherent were used as osteoclast precursor (BMMs). For generation of osteoclasts, the BMMs were seeded 10^6^ cells/well in 6-well plates with M-CSF (30 ng/mL) and 100 ng/mL RANKL followed by medium change every 3 days. After incubation for 7 days, differentiated BMMs were fixed with 4% paraformaldehyde and stained for tartrate-resistant acid phosphatase (TRAP). TRAP-positive multinucleate cells (TRAP+MNCs) containing more than three nuclei were counted as osteoclasts.

### 2.3. Alkaline Phosphatase (ALP) Staining

The differentiated C2C12 cells were washed with phosphate-buffered saline (PBS) and fixed with 10% formaldehyde for 15 min. After being rinsed with PBS again, cells were stained with the BCIP/NBT color development substrate (Sigma-Aldrich, St. Louis, MO, USA) for 30 min at RT to evaluate ALP activity. The ALP-positive cells were stained as purple or blue color [9].

### 2.4. Pit Formation Assay

Bone resorption activities were measured by bone resorption assay kit (Cosmo Bio, Tokyo, Japan). To avoid the interruption of fluorescence measurement, phenol red free *α* MEM (Thermo, Rochester, USA) containing 10% fetal bovine serum was added. BMMs were inoculated into the 48-well plate coated with fluoresceinamine-labeled chondroitin sulfate (FACS) and calcium phosphate. And then BMMs were incubated in conditioned medium containing RANKL 200 ng/mL and M-CSF 30 ng/mL for 6 days without medium change. The fluorescence of medium was evaluated by a fluorometric plate reader (SpectraMax i3, Molecular Device, CA, USA). Pit area was measured after the plate was washed with 5% sodium hypochlorite (Sigma-Aldrich, St. Louis, MO, Sigma).

### 2.5. Immunocytochemistry

Immunocytochemistry was performed to assess the effects of auranofin on the nuclear translocation of p65 in the Raw264.7 macrophage cell line. The Raw264.7 cells treated with vehicle or 3 *μ*M auranofin were fixed with 4% paraformaldehyde for 15 min, washed three times with 0.2% Triton X-100 in PBS for 10 min, blocked with 1% horse serum in PBS, and incubated with monoclonal anti-p65 antibody followed by Alexa-488 conjugated goat anti-mouse IgG antibody (A-21202, Invitrogen, Carlsbad, CA). Cells were counterstained with prolong gold antifade mountant with 4′,6-diamidino-2-phenylindole (DAPI, Thermo Scientific, Rochester, USA).

### 2.6. Animals and Experimental Design

8-week-old ICR female mice were obtained from Orient Bio Co. (Sungnam, Korea). The mice were acclimatized for at least 2 weeks prior to use and were housed in an air-conditioned room with a 12 h light/dark cycle at a temperature of 21 ± 2°C and 50 ± 5% humidity. The mice were anesthetized with tribromoethanol (Avertin) and either sham-operated (SHAM, controls; *n* = 6), or surgically bilaterally ovariectomized (OVX; *n* = 12). Thirteen weeks after surgery, the OVX mice were divided into two groups with 6 mice per group as follows: OVX control group and auranofin group. 10 mg/kg body weight auranofin suspended in 60% polyethylene glycol (PEG) was orally administered for 43 days. Mice were sacrificed at the end of treatment, and blood samples were obtained for serum biochemistry analysis. The collected distal femurs were fixed overnight in 75% ethanol prior to bone mass measurement [10]. The animal handlings and experimental procedures were performed in accordance with the regulations and rules of the Animal Ethics Committee of Sungkyunkwan University.

### 2.7. Serum Biochemistry Analysis

As a representative biochemical parameter relating to bone turnover, activity of bone alkaline phosphatase (BALP) was measured using commercial kit (MBS703336, MyBioSource, San Diego, CA) in accordance with the manufacturer's instructions.

### 2.8. Microcomputed Tomography Analysis

Bone morphometric parameters and microarchitectural properties were analyzed by microcomputed tomography (mCT) of the left femur using a SkyScan 1172 micro-CT scanner (Kontich, Belgium). *μ*CT images were scanned by using a high-resolution SkyScan 1172 system (SkyScan, Kontich, Belgium) at 50 kV and 201 *μ*A with a 0.5 mm aluminum filter and a resolution of 11 *μ*m pixel^−1^. Images were captured every 0.7° over an angular range of 180°. The bone mineral density (BMD), trabecular number (Tb.N), bone volume/total volume ratio (BV/TV), trabecular thickness (Tb.Th), trabecular pattern factor (Tb.Pf), and structure model index (SMI) were used to produce quantitative data in the SkyScan NRecon program and analyzed using SkyScan CTAN software.

### 2.9. Bone Histomorphometric Analysis

Collected tibias were fixed in 4% paraformaldehyde and decalcified in 10% EDTA buffer for 2 weeks at 4°C. Samples were gradually dehydrated and embedded in paraffin and cut into 4 mm-thick longitudinal sections. After the sections were deparaffinized with xylene, they were stained with hematoxylin and eosin (H&E) [11].

### 2.10. Western Blot Analysis

Proteins were separated by sodium dodecyl sulfate-polyacrylamide gel electrophoresis (SDS–PAGE), and the fractionated proteins were then transferred electrophoretically to nitrocellulose paper. Immune complexes were detected by using the Immobilon Western Chemiluminescent HRP Substrate (CB1001, Merck Millipore, Billerica, MA). Densitometric protein levels were quantified by the image software, Multi Gauge, V3.0 (Fujifilm, Tokyo, Japan).

### 2.11. Measurement of Cell Viability

MTT assay was used to assess the cell viability. Briefly, 100 *μ*L MTT (1 mg/mL diluted in phosphate-buffered saline) was added to each well of 96-well plates and the cells were incubated for 2 h. The culture media were removed by careful pipetting, and the resident cells were dissolved in 100 *μ*L DMSO. The absorbance was measured at 490 nm with a TriStar microplate reader (Berthold Technologies, Bad Wildbad, Germany).

### 2.12. Reporter Gene Assay

Lipofectamine 2000 (Invitrogen, Carlsbad, CA) was used for transient transfection. Raw264.7 cells were plated on 48-well plates 1 day prior to transfection. 70% confluent Raw264.7 cells were transfected with reporter plasmids (1 *μ*g pNF-*κ*B-luciferase and 0.3 *μ*g of pRL-SV40). The transfected cells were treated with 1 and 3 *μ*M auranofin for 24 h and then lysed with passive lysis buffer. Luciferase activities were determined by dual-luciferase reporter assay kit (E1960, Promega, Madison, MI), and firefly NF-*κ*B promoter activity was normalized to the corresponding *renilla* activity.

### 2.13. RNA Preparation and Real-Time Quantitative Polymerase Chain Reaction (qPCR)

Total RNA was isolated using TRIzol® reagent (Thermo Fisher Scientific, Waltham, MA, USA), and Oligo (dT) primers and reverse transcriptase (iNtRON Biotechnology, Seongnam, Korea) were used to synthesize cDNA. The mRNA expression levels of osteoclast markers, TRAP, and cathepsin K (CSTK) were quantified by real-time qPCR using a SYBR Select Master Mix (Applied Biosystems, Foster City, California) and Bio-Rad CFX Manager™ Software (Bio-Rad, Hercules, CA, USA). 18s rRNA protein mRNA was employed as a reference gene for normalizing mRNA levels. PCR was performed using selective primers for mouse TRAP (sense primer, 5′-GCAGTATCTTCAG GACGAGAAC-3′; antisense primer, 5′-TCCATAGTGAAACCGCAAGTAG-3′), CSTK (sense primer, 5′-CGAAAAGAGCCTAGC GAACA-3′; antisense primer, 5′-TGGGTAGCAGCAGAAACTTG-3′), and S18 ribosomal protein (S18r) genes (sense primer, 5′-GTAACCCGTTGAACCCCATT-3′; antisense primer, 5′-CCATCCAATCGGTAGTAGCG-3′).

### 2.14. Enzyme-Linked Immunosorbent Assay (ELISA)

Mouse interleukin-1*β* (IL-1*β*) ELISA kit (R&D Systems, Minneapolis, MN, USA) was used to measure concentration of IL-1*β* in culture media.

### 2.15. Statistical Analysis

Statistical significance was determined using a Student *t*-test, setting *P* < 0.05 as the level of significance. All experiments were carried out in triplicate at least.

## 3. Results

### 3.1. Inhibition of RANKL-Induced Osteoclast Differentiation by Auranofin

**Figure 1:**
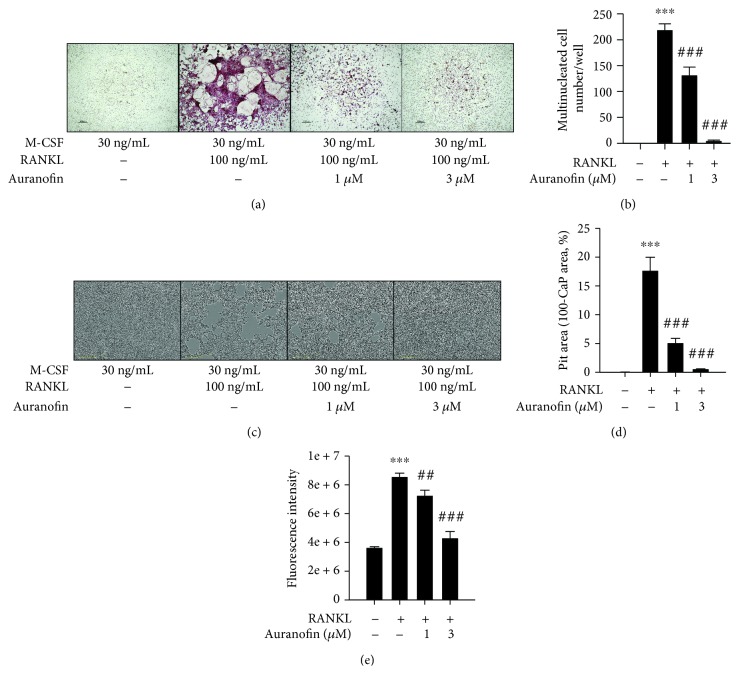
The inhibitory effect of auranofin on the resorption of CaP induced by RANKL. BMMs were isolated from C57BL/6N mice and cultured with 30 ng/mL M-CSF. After 3 days of incubation, adherent cells were incubated with 10% FBS/*α*-minimum essential medium containing 30 ng/mL M-CSF and 100 ng/mL RANKL and indicated the concentration of auranofin. For osteoclast formation, cells were stimulated with 100 ng/mL RANKL for 7 days. (a) Representative images of TRAP staining. (b) Number of TRAP-positive multinuclear cells (*n* = 3). (c) Representative images of pit formation assay on a CaP-coated plate. (d, e) The resorption activities were evaluated by fluorescence intensity and pit area. Data represents mean ± SD. ^∗∗∗^ *P* < 0.005 compared with control group; ^##^ *P* < 0.001 and ^###^ *P* < 0.005 compared with the RANKL-treated group.

Murine BMMs were used to test the antiosteoclastogenic activity of auranofin. BMMs were incubated with 100 ng/mL RANKL and 30 ng/mL M-CSF for 7 days, after which the cells differentiated into osteoclast-like giant and multinucleated tartrate-resistant acidic phosphatase- (TRAP-) positive cells (Figure 1(a)). The formation of TRAP-positive cells slowly diminished in BMMs incubated with 1 or 3 *μ*M auranofin (Figure 1(a)). Similar decreases in the number of multinucleated cells were also observed in response to auranofin in a concentration-dependent manner (Figure 1(b)). To assess whether the antiosteoclastogenesis leads to inhibition of bone resorption, BMMs were plated on a fluoresceinated calcium phosphate-coated plate and stimulated with M-CSF and RANKL in the presence or absence of auranofin. BMMs stimulated with M-CSF and RANKL formed a number of resorption pits, while the area of pits was significantly reduced by 1 and 3 *μ*M auranofin (Figures 1(c) and 1(d)). Moreover, the fluorescence intensity of medium resulting from RANKL-induced bone resorption activity was decreased by auranofin treatment (Figure 1(e)).

**Figure 2:**
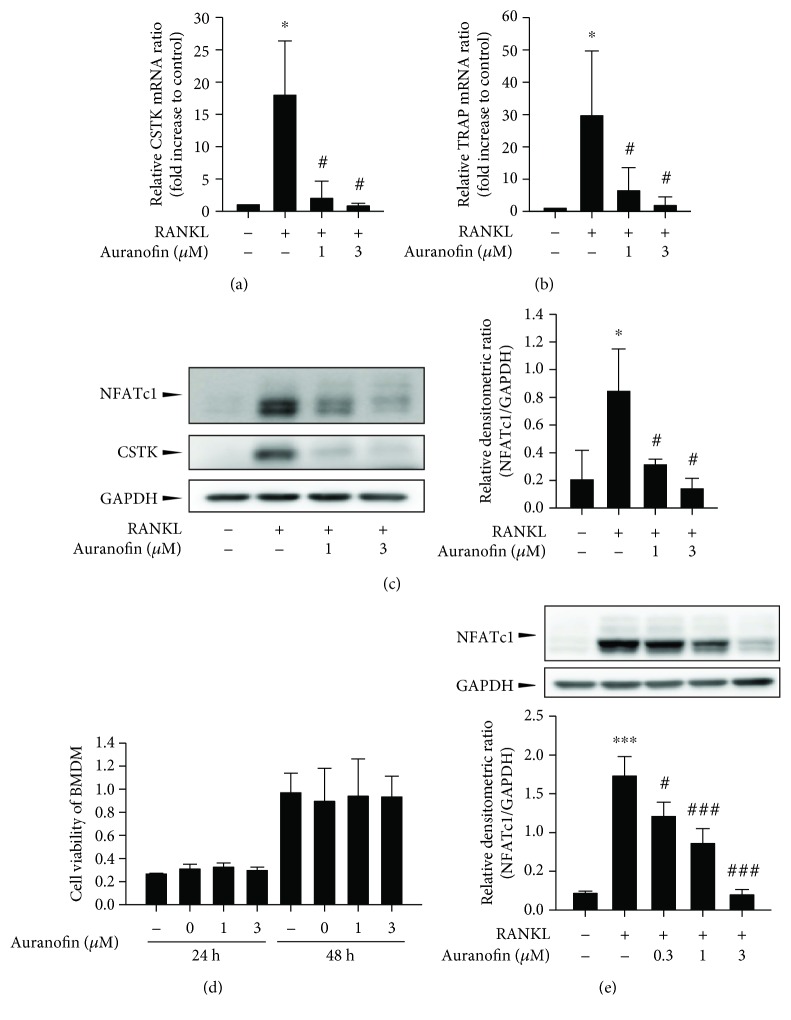
Inhibition of RANKL-induced osteoclast differentiation by auranofin. (a, b) mRNA levels of CSTK and TRAP (*n* = 4). mRNA levels were determined by real-time qPCR. (c) The protein expression level of NFATc1 and CSTK (*n* = 3). (d) Cytotoxicity of auranofin in BMMs (*n* = 6). MTT assays were performed to evaluate cytotoxicity. (e) Effect of auranofin on the protein expression of NFATc1 in RANKL-treated Raw264.7 cells (*n* = 3). Data represents mean ± SD; ^∗^ *P* < 0.05 and ^∗∗∗^ *P* < 0.005 compared with the control group; ^#^ *P* < 0.05 and ^###^ *P* < 0.005 compared with the RANKL-treated group.

Those observations were further supported by real-time qPCR. Auranofin (1 and 3 *μ*M) almost completely inhibited RANkKL-induced mRNA expression of CSTK and TRAP, two osteoclast-selective markers (Figures 2(a) and 2(b)). RANKL-induced activation of nuclear factor of activated T-cells1 (NFATc1) is essential for achieving terminal differentiation to osteoclasts [12]. To test the effects of RANKL on osteoclast differentiation, BMMs were exposed to RANKL with and without auranofin and screened for NFATc1 and CSTK protein expression. Treatment with 1 and 3 *μ*M auranofin potently suppressed RANKL-mediated expression of both NFATc1 and CSTK (Figure 2(c)). Next, an MTT assay was carried out to exclude the possibility that the inhibition of osteoclastogenesis by auranofin is due to nonspecific cytotoxicity. No cytotoxicity was evident in BMDMs treated with 0.3–3 *μ*M auranofin (Figure 2(d)). We further confirmed that auranofin (0.3–3 *μ*M) inhibited NFATc1 expression in a concentration-dependent manner using RANKL-exposed Raw264.7 macrophages, a surrogate cell line of BMMs (Figure 2(e)). Treatment of Raw264.7 cells with 0.3–3 *μ*M auranofin for 48 h marginally affected cell viability (data not shown).

### 3.2. Effect of Auranofin on RANKL-Activated IKK/I*κ*B*α*

**Figure 3:**
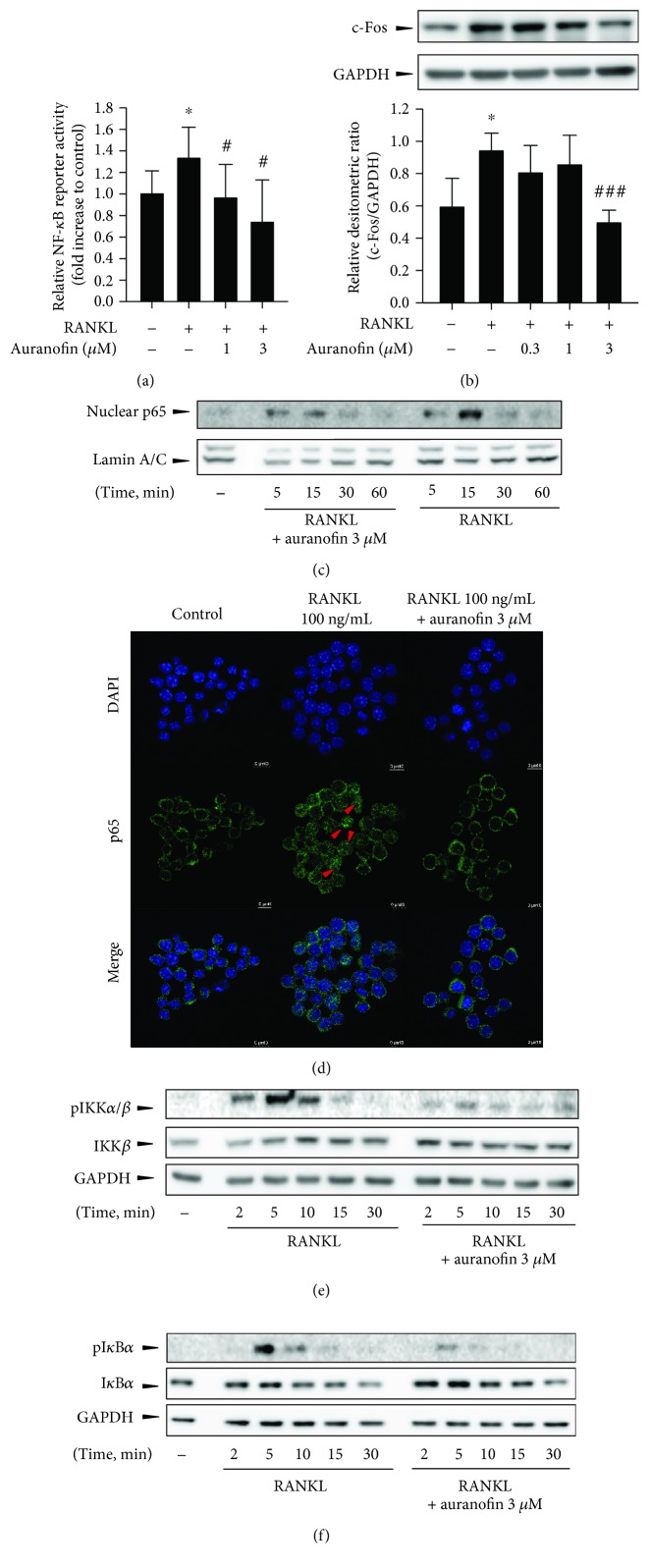
Effects of auranofin on RANKL-activated IKK/I*κ*B*α*–dependent NF-*κ*B activation. (a) Relative activity of the NF-*κ*B reporter gene in 100 ng/mL RANKL-exposed Raw264.7 cells (*n* = 6). (b) Effect of auranofin on c-Fos expression in BMMs (*n* = 3). (c, d) Effect of auranofin on nuclear translocation of p65. BMMs were preincubated with 3 *μ*M auranofin for 15 min and exposed to 100 ng/mL RANKL for the indicated time points. Nuclear translocation of p65 was visualized by immunoblottings using subcellular fractionation (c) and immunocytochemistry (d, red arrows indicate nuclear p65). (e, f) Effects of auranofin on phosphorylation of IKK*α*/*β* and I*κ*B*α* in RANKL-stimulated BMMs. ^∗^ *P* < 0.05 compared with the control group; ^#^ *P* < 0.05 and ^###^ *P* < 0.005 compared with the RANKL-treated group.

Transcription of NFATc1 is mainly controlled by NF-*κ*B [12]; however, the AP-1 family, including c-Fos, has been shown to trigger a transcriptional regulatory cascade by producing and cooperating with NFATc1. Furthermore, mice lacking c-Fos are osteoporotic because of a lack of osteoclast differentiation [13]. To investigate whether the inhibition of NF-*κ*B activity by auranofin leads to blockade of osteoclast differentiation, we performed reporter gene analyses using a pNF-*κ*B-luciferase reporter. In Raw264.7 cells transiently transfected with a luciferase reporter construct containing NF-*κ*B-binding regions, auranofin significantly inhibited RANKL-induced NF-*κ*B luciferase reporter activity (Figure 3(a)). Although c-Fos plays a pivotal role in osteoclast differentiation, enhanced c-Fos expression by RANKL was only moderately affected by auranofin treatment in Raw264.7 cells, although significant decreases were observed in cells treated with the highest dose (3 *μ*M) of auranofin (Figure 3(b)).

As low concentrations of auranofin were found to effectively inhibit NF-*κ*B activity and NFATc1 expression in osteoclast precursor cells, we next focused on how auranofin suppresses RANKL-induced NF-*κ*B activation. Since p65 is a major transcription factor for NF-*κ*B activation by RANKL, we examined nuclear translocation of p65 by subcellular fractionation with immunoblotting. Nuclear p65 protein levels increased from 5 to 15 min after treatment with RANKL (100 ng/mL), with 3 *μ*M auranofin substantially inhibiting nuclear translocation of p65 at 15 min after RANKL treatment (Figure 3(c)). Immunocytochemistry using a p65 antibody confirmed that the majority of p65 was localized in the cytoplasm of quiescent Raw264.7 cells (Figure 3(d)). After stimulus with RANKL for 15 min, nuclear p65 was detected in some populations of Raw264.7 cells (Figure 3(d)); however, the nuclear translocation of p65 was completely blocked by 3 *μ*M auranofin (Figure 3(d)).

Next, we assessed the effects of auranofin on upstream signaling pathways of NF-*κ*B in RANKL-treated Raw264.7 cells. Like most members of the TNF receptor superfamily, RANK engages TNF receptor-associated factors (TRAFs) to activate several kinase cascades including IKK-dependent I*κ*B*α* phosphorylation [14]. Under the control of TRAF6, transforming growth factor-*β*- (TGF-*β*-) activated kinase 1- (TAK1-) mediated phosphorylation of IKK plays a central role in activating the NF-*κ*B pathway in response to inflammatory cytokines and RANKL [15]. Compared to Raw264.7 cells treated with RANKL only, coincubation with 3 *μ*M auranofin led to significant reductions in IKK*β* phosphorylation (Figure 3(e)). TAK1-dependent IKK complex activation is followed by the phosphorylation and subsequent degradation of the I*κ*B*α* subunit [16]. Immunoblot analyses using specific antibodies confirmed that the phosphorylation and degradation of I*κ*B*α* by RANKL were also prevented by 3 *μ*M auranofin (Figure 3(f)). These data strongly support the notion that NFATc1 downregulation by auranofin may result from the inhibition of TAK1/IKK/I*κ*B*α* axis-dependent NF-*κ*B activation.

### 3.3. Inhibition of Inflammasome-Mediated IL-1*β* Secretion by Auranofin

IL-1*β*, one of the major proinflammatory cytokines, is known to promote RANKL expression in marrow stromal cells and directly induces osteoclastogenesis [17]. IL-1*β* is expressed by hematopoietic cells as a 31 kDa precursor, primarily in response to inflammatory stimuli. However, the full-length pro-IL-1*β* is inactive and must be cleaved into its mature 17 kDa form before binding to the IL-1 receptor [18]. In BMDMs, IL-1*β* secretion is thought to occur via a two-step process. The first signal can be triggered by various pathogen-associated molecular patterns (PAMPs) after Toll-like receptor (TLR) activation, resulting in NF-*κ*B-dependent synthesis of NLRP3 and pro-IL-1*β*. The second signal is mediated by activation of the inflammasome, resulting in cleavage of IL-1*β* by caspase-1 [19].

**Figure 4:**
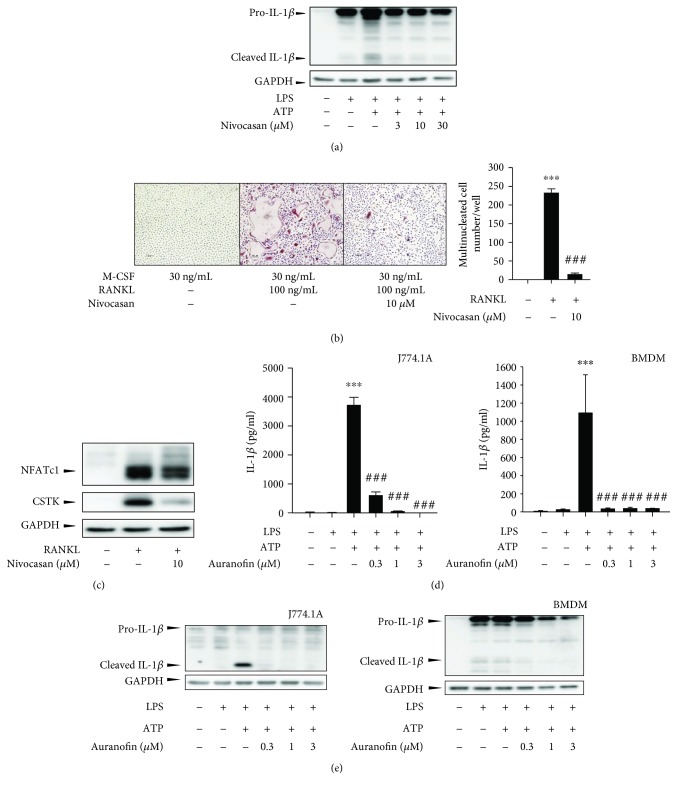
Inhibition of inflammasome-mediated IL-1*β* secretion by auranofin. (a) Effects of auranofin on the protein expression of pro-IL-1*β* and cleaved IL-1*β*. BMMs were primed with LPS (100 ng/mL) for 4 h and further incubated with 1 mM ATP and nivocasan (3-30 *μ*M) for 1 h. (b) Effect of the inflammasome inhibitor (nivocasan) on osteoclast differentiation. The left panel shows the representative images of TRAP stainings, and the number of TRAP-positive multinuclear cells was illustrated in the right panel (*n* = 3). (c) Effects of nivocasan on NFATc1 and c-Fos expression in RANKL-treated BMMs. (d) IL-1*β* ELISA results (*n* = 3). The secreted IL-1*β* levels were determined in culture media from J774.1A cells and BMDMs incubated with 1 mM ATP and auranofin (0.3-3 *μ*M) for 1 h followed by preincubation with 100 ng/mL LPS for 4 h. (e) Cell lysates from J774.1A cells (left) and BMDMs (right) were analyzed for protein expression of pro-IL-1*β* and cleaved IL-1*β* by immunoblottings. ^∗∗∗^ *P* < 0.005 compared with the control group; ^###^ *P* < 0.005 compared with the RANKL-treated group.

To test whether inflammasome-dependent IL-1*β* secretion is involved in the differentiation of BMMs to osteoclasts, we examined the effect of nivocasan (GS-9450), a pan-caspase inhibitor, on osteoclastogenesis. Nivocasan irreversibly arrests caspase-1 activity and inhibits that of caspase-8, both of which play a key role in IL-1*β* secretion [20]. BMDMs were exposed to lipopolysaccharide (LPS) and ATP to induce NLRP3 inflammasome-dependent IL-1*β* secretion. As shown in Figure 4(a), 10 *μ*M nivocasan treatment inhibited cleavage of pro-IL-1*β* into the active p17 fragment. The inhibition of IL-1*β* cleavage by nivocasan resulted in blockade of osteoclast differentiation, as evidenced by a decrease in the number of TRAP-positive and multinucleated cells (Figure 4(b)). Furthermore, protein expression of NFATc1 and CSTK was suppressed by nivocasan (Figure 4(c)). These results indicate that inflammasome signaling is actively involved in the differentiation of BMMs to osteoclasts.

As auranofin inhibits IL-1*β* secretion in the differentiated cells from BMMs [21], we hypothesized that auranofin may block osteoclast differentiation by interfering with inflammasome-mediated IL-1*β* secretion in BMDMs. In a manner similar to that of nivocasan, auranofin impairs inflammasome mediated IL-1*β* secretion in BMDM and J774.A1 cells (ASC-positive mouse monocyte/macrophage cell line) (Figure 4(d)). Western blot analyses confirmed that 0.3–3 *μ*M auranofin potently reduced the amount of cleaved IL-1*β* in both BMDM and J774.A1 cells exposed to LPS and ATP (Figure 4(e)). Interestingly, the inhibitory effects of auranofin on inflammasome activation were clearly observed at very low concentrations (0.3 and 1 *μ*M), suggesting that the antiosteoclastogenesis effect of auranofin is partly dependent on its ability to inhibit inflammasome activation.

### 3.4. Inhibition of Osteoporosis by Auranofin in Ovariectomized Mice

**Figure 5:**
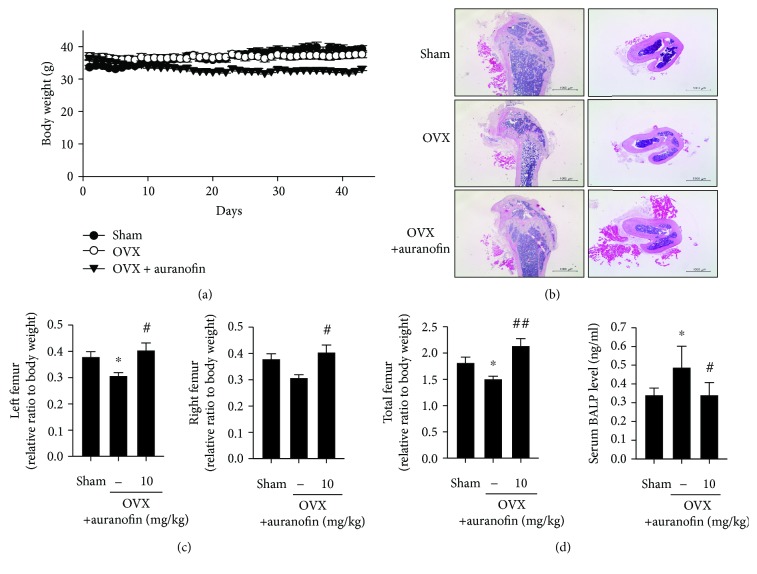
Inhibitory effect of auranofin on bone loss in ovariectomized mice. (a) Body weight change (*n* = 6 for each group). (b) H&E staining of the vertical and horizontal sections of femurs. (c) Weight of left, right, and total femur relative to body weight (*n* = 6) (d) Serum levels of BALP (*n* = 4). ^∗^ *P* < 0.05 compared with the control group; ^#^ *P* < 0.05 and ^##^ *P* < 0.01 compared with the RANKL-treated group.

Finally, we investigated the effects of auranofin on bone loss *in vivo* using an ovariectomized (OVX) mouse model of osteoporosis. OVX mice were orally administered with vehicle or 10 mg/kg auranofin for 43 days. The body weight of mice was measured daily for 43 days, with a final reading performed just before sacrifice. Body weights of the OVX and control groups treated with 10 mg/kg auranofin were virtually identical prior to initiation of the study. However, the total body weight of the auranofin-treated mice gradually decreased over the course of the study, supporting a previous finding that the major gastrointestinal side effect of auranofin is diarrhea [22] (Figure 5(a)).

As evidenced by representative H&E stains, auranofin administration inhibited trabecular bone loss in OVX mice (Figure 5(b)). Auranofin treatment almost completely restored weight loss in the left or right femur of OVX mice after 43 days (Figure 5(c)), whereas serum levels of bone alkaline phosphatase (BALP) in OVX mice were significantly higher than those of the sham-operated animals. BALP is released in the circulation during the bone-forming phase of the remodeling process [23]. Compared to OVX mice, auranofin-administrated mice showed decreased levels of serum BALP (Figure 5(d)).

**Figure 6:**
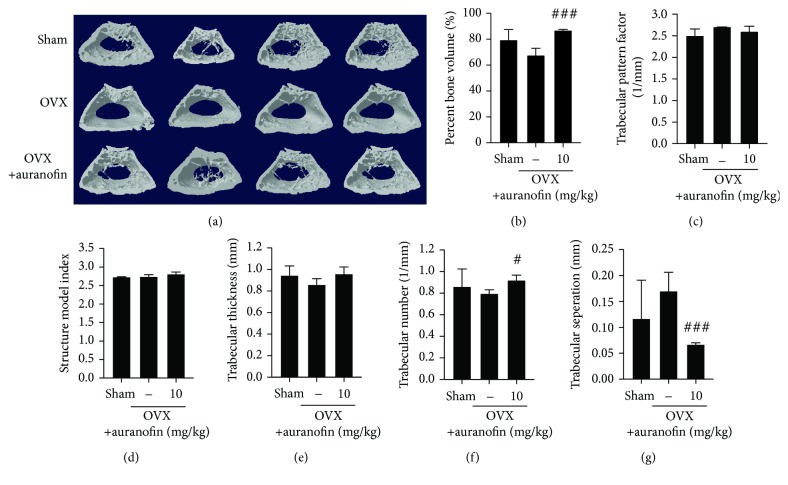
Micro-CT analyses. (a) Representative *μ*-CT three-dimensional reconstructed images from the fixed vertebral bodies of OVX mice. (b–g) Percent bone volume, trabecular pattern factor, structure model index, trabecular thickness, trabecular number, and trabecular separation obtained by analyzing the three mouse groups were presented (*n* = 6). ^#^ *P* < 0.05 and ^###^ *P* < 0.005 compared with the RANKL-treated group.

Results from these experiments were further analyzed by microcomputed tomography (micro-CT). Although calibrated BMD marked no significant decrease in OVX mice (data not shown), representative pictures of micro-CT sections showed improvement in bone density in auranofin-administered mice (Figure 6(a)). Auranofin suppressed the two- and three-dimensional structural bone loss in the OVX mice, as evidenced by changes in BMD, percent bone volume, trabecular thickness, and number (Figures 6(b) and 6(e)–6(g)). Although trabecular pattern factors and structural model indices showed no changes, the trabecular separation number was much lower in auranofin-administered mice, demonstrating the utility of auranofin for prevention of bone loss.

## 4. Discussion

Drug repositioning is an increasingly popular strategy in drug development, as it has the potential to both speed up the discovery of novel treatments and reduce the risk of drug failure. Many pharmaceutical companies are scanning the existing pharmacopoeia for repositioning candidates and the number of repositioning success stories is increasing [24]. Auranofin, which is clinically approved for use in RA therapy, is one such candidate, with this agent having been investigated for potential therapeutic application in a wide range of diseases, including cancer [25, 26].

Gold-based compounds, such as auranofin, are known to exhibit anti-inflammatory activity via the downregulation of NF-*κ*B [27] and may also exert effects on upstream targets of IKK-dependent I*κ*B*α* phosphorylation [28]. Based on previous studies demonstrating an essential role of NF-*κ*B activation in osteoclastogenesis [3, 29], we hypothesized that auranofin may block osteoclast differentiation. Here, we showed that auranofin treatment led to reduced differentiation of bone marrow cells to osteoclasts by inhibiting RANKL-induced osteoclast differentiation in Raw264.7 cells and BMMs. Under normal circumstances, osteoclast differentiation is mediated by the binding of RANKL to its target receptor, activating multiple intracellular signaling pathways including I*κ*B*α* kinase and NF-*κ*B [14]. The RANKL-induced phosphorylation of IKK/I*κ*B*α* was suppressed by auranofin, followed by reduced NF-*κ*B activation.

Auranofin also inhibited secretion of IL-1*β* from BMDMs. In an *in vitro* osteoclastogenesis assay using a pan-caspase inhibitor, we observed a close correlation between IL-1*β* secretion and osteoclast differentiation. Mature IL-1*β* is secreted via inflammasome-mediated activation and is responsible for osteoclast-induced bone resorption [17]. IL-1 was shown to directly induce osteoclast differentiation in BMMs expressing the IL-1 receptor via a RANKL-independent pathway [21]. In this study, we demonstrated that auranofin dramatically blocks IL-1*β* secretion from BMDMs. The antiosteoclastogenic activity of auranofin was further confirmed *in vivo* using an OVX mouse model of osteoporosis. Oral administration of 10 mg/kg auranofin led to significant recovery of weight loss in the femur of OVX mice. That inhibitory effect is thought to be closely correlated with the antiosteoclastogenic activity of auranofin as it showed no effect on bone morphogenetic protein- (BMP-) induced osteoblastic differentiation of C2C12 cells (Supplementary Figure 1). Serum BALP levels, which are indicative of bone turnover activity, were also reduced in OVX mice treated with auranofin. Furthermore, data from our micro-CT analyses strongly supported the results of *in vitro* assays examining anti-bone resorptive activity.

Taken together, the *in vitro* and *in vivo* results presented here clearly support the use of auranofin as a potent inhibitor of BMM differentiation to osteoclasts. Denosumab is the first drug targeting osteoclast differentiation and has been approved for the treatment of malignant osteoporosis in both the United States and Europe. Despite its high efficacy and low rate of adverse events in clinical trials [30], its high cost and inconvenient parenteral injection have led to continued interest in alternative therapeutic options. Here, we propose auranofin as a potential substitute for denosumab, as it is cost-effective and orally available. Moreover, auranofin has undergone extensive toxicological analyses and has proven safe for human use.

RANKL, through its ability to stimulate osteoclast formation and activity, is a critical mediator of bone resorption and overall bone density. Overproduction of RANKL is implicated in a variety of degenerative bone diseases, including RA and psoriatic arthritis. In RA, RANKL is expressed by both T cells and synoviocytes, leading to persistent osteoclast activation and bone erosion [31]. The data presented here further suggest that auranofin exerts anti-RA activity via targeting of RANKL-induced osteoclast maturation.

## 5. Conclusion

The current results suggested that auranofin could serve as a novel inhibitor of osteoclastogenesis by suppressing IKK-I*κ*B*α* phosphorylation and inflammasome-mediated IL-1*β* secretion. Based on these findings, auranofin represents an excellent candidate for drug repurposing, offering potential therapeutic efficacy for osteoclast-related disorders outside of its approved use in RA.

## Data Availability

The data used to support the findings of this study are available from the corresponding author upon request.

## Supplementary Materials

Supplementary Materials Supplementary Figure 1: alkaline phosphatase (ALP) staining. Osteoblast differentiation of C2C12 cells was induced by bone morphogenetic protein (BMP). ALP staining was performed to evaluate the effect of auranofin on the differentiation capacity.

## References

[1] Manolagas S. C. (2000). Birth and death of bone cells: basic regulatory mechanisms and implications for the pathogenesis and treatment of osteoporosis. *Endocrine Reviews*.

[2] Martin T. J., Sims N. A. (2005). Osteoclast-derived activity in the coupling of bone formation to resorption. *Trends in Molecular Medicine*.

[3] Boyle W. J., Simonet W. S., Lacey D. L. (2003). Osteoclast differentiation and activation. *Nature*.

[4] Rachner T. D., Khosla S., Hofbauer L. C. (2011). Osteoporosis: now and the future. *The Lancet*.

[5] Silverman S., Agodoa I., Kruse M., Parthan A., Orwoll E. (2015). Denosumab for elderly men with osteoporosis: a cost-effectiveness analysis from the US payer perspective. *Journal of Osteoporosis*.

[6] Yanni G., Nabil M., Farahat M. R., Poston R. N., Panayi G. S. (1994). Intramuscular gold decreases cytokine expression and macrophage numbers in the rheumatoid synovial membrane. *Annals of the Rheumatic Diseases*.

[7] Jeon K.-I., Jeong J.-Y., Jue D.-M. (2000). Thiol-reactive metal compounds inhibit NF-*κ*B activation by blocking I*κ*B kinase. *The Journal of Immunology*.

[8] Hall T. J., Jeker H., Nyugen H., Schaeublin M. (1996). Gold salts inhibit osteoclastic bone resorption in vitro. *Inflammation Research*.

[9] Han Y., Kim M. J., Lee K. Y. (2018). Berberine derivative, Q8, stimulates osteogenic differentiation. *Biochemical and Biophysical Research Communications*.

[10] Choi J. H., Han Y., Kim Y. A. (2017). Platycodin D inhibits osteoclastogenesis by repressing the NFATc1 and MAPK signaling pathway. *Journal of Cellular Biochemistry*.

[11] Lee J., Youn B. U., Kim K. (2015). Mst2 controls bone homeostasis by regulating osteoclast and osteoblast differentiation. *Journal of Bone and Mineral Research*.

[12] Takayanagi H., Kim S., Koga T. (2002). Induction and activation of the transcription factor NFATc1 (NFAT2) integrate RANKL signaling in terminal differentiation of osteoclasts. *Developmental Cell*.

[13] Matsuo K., Galson D. L., Zhao C. (2004). Nuclear factor of activated T-cells (NFAT) rescues osteoclastogenesis in precursors lacking c-Fos. *Journal of Biological Chemistry*.

[14] Ruocco M. G., Maeda S., Park J. M. (2005). I*κ*B kinase (IKK) *β*, but not IKK*α*, is a critical mediator of osteoclast survival and is required for inflammation-induced bone loss. *Journal of Experimental Medicine*.

[15] Huang H., Ryu J., Ha J. (2006). Osteoclast differentiation requires TAK1 and MKK6 for NFATc1 induction and NF-*κ*B transactivation by RANKL. *Cell Death & Differentiation*.

[16] Devi Menon S., Guy G. R., Tan Y. H. (1995). Involvement of a putative protein-tyrosine phosphatase and I*κ*B-*α* serine phosphorylation in nuclear factor *κ*B activation by tumor necrosis factor. *Journal of Biological Chemistry*.

[17] Wei S., Kitaura H., Zhou P., Ross F. P., Teitelbaum S. L. (2005). IL-1 mediates TNF-induced osteoclastogenesis. *Journal of Clinical Investigation*.

[18] Perregaux D., Gabel C. A. (1994). Interleukin-1 beta maturation and release in response to ATP and nigericin. Evidence that potassium depletion mediated by these agents is a necessary and common feature of their activity. *Journal of Biological Chemistry*.

[19] Afonina I. S., Müller C., Martin S. J., Beyaert R. (2015). Proteolytic processing of interleukin-1 family cytokines: variations on a common theme. *Immunity*.

[20] Kudelova J., Fleischmannova J., Adamova E., Matalova E. (2015). Pharmacological caspase inhibitors: research towards therapeutic perspectives. *Journal of Physiology and Pharmacology*.

[21] Kim J. H., Jin H. M., Kim K. (2009). The mechanism of osteoclast differentiation induced by IL-1. *The Journal of Immunology*.

[22] Behrens R., Devereaux M., Hazleman B., Szaz K., Calvin J., Neale G. (1986). Investigation of auranofin-induced diarrhoea. *Gut*.

[23] Pedrazzoni M., Alfano F. S., Girasole G. (1996). Clinical observations with a new specific assay for bone alkaline phosphatase: a cross-sectional study in osteoporotic and pagetic subjects and a longitudinal evaluation of the response to ovariectomy, estrogens, and bisphosphonates. *Calcified Tissue International*.

[24] Ashburn T. T., Thor K. B. (2004). Drug repositioning: identifying and developing new uses for existing drugs. *Nature reviews Drug Discovery*.

[25] Roder C., Thomson M. J. (2015). Auranofin: repurposing an old drug for a golden new age. *Drugs in R&D*.

[26] Fiskus W., Saba N., Shen M. (2014). Auranofin induces lethal oxidative and endoplasmic reticulum stress and exerts potent preclinical activity against chronic lymphocytic leukemia. *Cancer Research*.

[27] Youn H. S., Lee J. Y., Saitoh S. I., Miyake K., Hwang D. H. (2006). Auranofin, as an anti-rheumatic gold compound, suppresses LPS-induced homodimerization of TLR4. *Biochemical and Biophysical Research Communications*.

[28] Jeon K.-I., Byun M.-S., Jue D.-M. (2003). Gold compound auranofin inhibits I*κ*B kinase (IKK) by modifying Cys-179 of IKK*β* subunit. *Experimental & Molecular Medicine*.

[29] Jimi E., Aoki K., Saito H. (2004). Selective inhibition of NF-*κ*B blocks osteoclastogenesis and prevents inflammatory bone destruction in vivo. *Nature Medicine*.

[30] Dempster D. W., Lambing C. L., Kostenuik P. J., Grauer A. (2012). Role of RANK ligand and denosumab, a targeted RANK ligand inhibitor, in bone health and osteoporosis: a review of preclinical and clinical data. *Clinical Therapeutics*.

[31] Firestein G. S. (2003). Evolving concepts of rheumatoid arthritis. *Nature*.

