# Supplementary material for: Auranofin Inhibits RANKL-Induced Osteoclastogenesis by Suppressing Inhibitors of κB Kinase and Inflammasome-Mediated Interleukin-1β Secretion
Source: Oxid Med Cell Longev. 2019 Apr 22;2019:3503912. doi: 10.1155/2019/3503912 (PMC6561666; doi:10.1155/2019/3503912)
Supplement: Supplementary Materials — Supplementary Figure 1: alkaline phosphatase (ALP) staining. Osteoblast differentiation of C2C12 cells was induced by bone morphogenetic protein (BMP). ALP staining was performed to evaluate the effect of auranofin on the differentiation capacity. [file 3503912.f1.pdf]

Supplementary figure 1

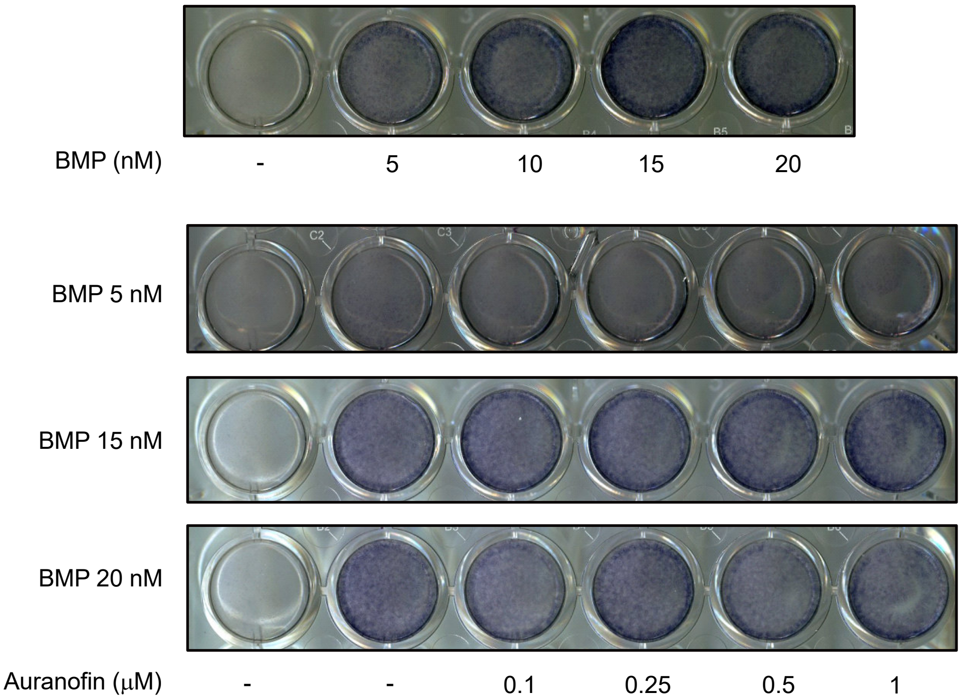

Supplementary figure 1. Alkaline phosphatase (ALP) staining. Osteoblast differentiation of C2C12 cells were induced by bone morphogenetic protein (BMP) . ALP staining were performed to evaluate the effect of auranofin on the differentiation capacity.
